# Bactericidal Effect of Entomopathogenic Bacterium *Pseudomonas entomophila* Against *Xanthomonas citri* Reduces Citrus Canker Disease Severity

**DOI:** 10.3389/fmicb.2020.01431

**Published:** 2020-06-24

**Authors:** Sonia Villamizar, Jesus Aparecido Ferro, Juan Carlos Caicedo, L. M. C. Alves

**Affiliations:** ^1^Post Graduate Program in Agricultural and Livestock Microbiology, School of Agricultural and Veterinary Sciences, São Paulo State University (UNESP), Jaboticabal, Brazil; ^2^Faculty of Exact, Natural and Agricultural Sciences, Research Group CIBAS, Universidad de Santander (UDES), Bucaramanga, Colombia

**Keywords:** biological control, anti bacterial, NRPS, secondary metabolites, antagonism ability, *Citrus limonia*

## Abstract

The bacterium *Pseudomonas entomophila* has been recognized as an exceptional species within the *Pseudomonas* genus, capable of naturally infecting and killing insects from at least three different orders. *P. entomophila* ingestion leads to irreversible gut damage resulting from a global blockage of translation, which impairs both immune and tissue repair systems in the insect intestine. In this study we isolated a *P. entomophila* bacterial strain from soil samples which displayed a strong activity against *Xanthomonas citri* subsp, *citri* (*Xcc*), the etiological agent of citrus canker disease. The antagonism potential of isolated bacteria against *Xcc* and its ability to reduce citrus canker severity was assessed both *ex planta* and *in planta*. Our findings show that pathogenicity assays in *Citrus x limonia* by pressure infiltration and spray with a mixture of *P. entomophila* and *Xcc* leaded to a significant reduction in the number of canker lesions in high susceptible citrus leaves, at 21 days post-infection. To the best of our knowledge this is the first report of antibacterial activity of *P. entomophila* against a phytopathogenic bacterium. Collective action of *P. entomophila* factors such as diketopiperazine production and the type 6 secretion system (T6SS) may be involved in this type of biological control of citrus canker. The results suggest that the *P. entomophila* strain could be a promising biocontrol agent acting directly against *Xcc.*

## Introduction

Sustained world population growing has forced farmers to implement unfriendly practices toward the environment (i.e., arbitrary use of pesticides and chemical fertilizers) to attend to rising demand for higher yields of agricultural products. The increasing gap between supply and demand and the unfavorable impact on the environment has encouraged researchers to develop alternative strategies, searching to promote a sustainable agriculture. Several genera of both soil and plant associated bacteria became in powerful tools in sustainable agriculture, since these bacteria display extremely versatile secondary metabolisms, which provide useful sources of many diverse metabolites with valuable biological activities, including antibiotic activity (Raaijmakers and Mazzola, 2012).

*Pseudomonas* is a bacterial genus characterized by a great environmental ubiquity not only due to the extraordinary ability of members to produce a wide diversity of secondary metabolites (e.g., phenazines, pyoluteroina or lipopeptides), but also in their use of a wide diversity of organic molecules as an energy source (Wu et al., 2010). Some *Pseudomonas* species are phytopathogens producing leaf-spot, wilt and blight. Others have the capacity to establish themselves as natural inhabitants of the rhizosphere and phyllosphere, acting as commensals or providing beneficial activity to their host. They can promote suppression of pathogen infection and/or directly facilitate host growth and fitness (Mercado-Blanco and Bakker, 2007).

Beneficial *Pseudomonas* species display features that enable them to act as effective biological control agents (BCAs) against several phytopathogens. Among these attributes the usually shared by a widespread range of *Pseudomonas* strains are: (I) Pronounced colonizing ability of plant surfaces, internal plant tissues and phytopathogen structures (e.g., hyphae) (Mercado-Blanco and Bakker, 2007); (II) The capacity for production of several types of antibiotic which provides an additional advantage in competition with local microbiota and phytopathogens (Gross and Loper, 2009); and (III) The ability to trigger resistance responses in host plants (Djavaheri et al., 2012). Therefore, strategies of direct antagonism as antibiotic production or indirect approaches such as competition for nutrients (e.g., siderophore production), besides to induction of systemic resistance responses, actively participate in phytopathogenic disease suppression by the Pseudomonads (Zabiha et al., 2011). The *Pseudomonas* strains best renowned for their biocontrol activity against phytopathogenic microorganisms are: *Pseudomonas fluorescens*, *Pseudomonas protegens*, *Pseudomonas Chlororaphis*, and *Pseudomonas putida* (Gu and Mazzola, 2001; Ramette et al., 2011).

Recent studies have shown that cooperation among bacterial species is relatively rare and competition is the dominant relationship between microbes that share an ecological niche (Foster and Bell, 2012). Because the Gammaproteobacteria *Pseudomonas* and *Xanthomonas*, have been the bacterial genera most consistently detected by metagenomic approaches in several phyllospheres (Vorholt, 2012). it is reasonable to think that there might be a competitive relationship between these two bacterial genera in this environmental niche where resource availability is greatly reduced. In accordance with this and the findings of our previous study (Meneghine et al., 2017; Villamizar, 2017), carried out using a whole metagenomics sequencing approach of soil samples from São Paulo Zoo, it which shown genes that encode several metabolites with antibacterial activity, which were mainly associated to genome of gamma-proteobacteria group specially from *Pseudomonadaceae* family, we suggest that Pseudomonads bacteria recovered from soil samples from São Paulo Zoo could display antagonism against *Xanthomonas citri* subsp. *citri* 306 (*Xcc* 306), the etiological agent of citrus canker disease. This disease affecting almost all types of citrus crops. *Xcc* 306 inducing cell hyperplasia, leading to rupture of the leaf epidermis and resulting in raised corky and spongy lesions surrounded by a water-soaked margin, i.e., the pathognomonic canker lesion (Schaad et al., 2006). Disease management has been based in both; tree eradication and copper spray treatment. Overuse of copper for control of bacterial citrus canker has led to the development and prevalence of copper-resistant strains of *Xcc* (Behlau et al., 2011).

The main goals of this study were: to assess *ex planta* and *in planta*, the antagonistic potential of isolated bacteria from soil samples from São Paulo Zoo against *Xcc* 306, likewise, to evaluate its ability to reduce canker disease severity in a high susceptible citrus host, in order to provide an alternative to traditional copper treatment which has significant environmental implications. In this study coupling metagenomic and culture approaches, we have isolated and identified a bacterial strain with 99.5% 16S rDNA gene sequence similarity with *Pseudomonas entomophila* from soil samples. This strain displayed strong bactericidal activity against *Xcc* 306, besides, shown a great reduction of canker lesions in a susceptible citrus host. To the best of our knowledge, there is currently no information regarding potential bactericidal activity of *P. entomophila* against phytopathogenic bacteria. Most studies of *P. entomophila* have focused on its entomopathogenicity ability, since, it is capable of naturally infecting and killing insects from at least three different orders.

## Materials and Methods

### Soil Sampling

In the present study 12 samples were used. These were been taken at a depth of between 0 and 20 cm from the São Paulo Zoo Park farm, São Paulo, Brazil, from soil which has been supplemented with organic compost in the last 5 years. The compost was made at the São Paulo Zoo Park composting facility from organic waste such as: tree branches, leaves, grass, manure, bedding, and food residues from about 400 animal species inhabitants of the zoo mammals, avian and reptiles.

### Metagenomic DNA Extraction, Sequencing and Functional Screening

In order to corroborate the presence in the soil samples of genes encoding metabolites with recognized bactericidal activity, which were identified in a previous study, a metagenomic approach was performed. Briefly, Metagenomic DNA was extracted from 0.25 g soil samples using Powersoil^®^ DNA isolation kit (Mo Bio Laboratories, Carlsbad, CA, United States), according to the manufacturer’s instructions. DNA quality was evaluated by 260 nm/280 nm, and 260 nm/230 nm ratios. Final DNA concentrations were measured by Qubit 3.0 flourometer using Qubit^®^ dsDNA BR Assay Kit. A metagenomic DNA paired-end 100 nt library was constructed using the Illumina^®^ TruSeq^®^ DNA Sample Preparation Kits following the manufacturer’s instructions. After quality filtering by Scythe, Cutadapt and PrinSeq, unassembled reads were submitted for analysis and functional screening on the MG-RAST metagenomics analysis server (Meyer et al., 2008). Our findings were compared with the COG and SEED Subsystem databases to assess the prominent functions of major bacterial genera in samples.

### Isolation and Characterization of Pseudomonads

To recover bacterial species of the *Pseudomonas* genus from soil samples, selective and differential microbiological media culture were used. 500 mg of each soil sample were homogenized in 5 ml of 10 mM phosphate buffer pH 7.0. Three ten-fold dilution series of this suspension were spread on modified King’s B agar (Peptone 20 g/L, dipotassium hydrogen phosphate 1.5 g/L, magnesium sulfate 1.5 g/L, agar 15 g/L, and 5-Chloro-2-(2,4-dichlorophenoxy) phenol 25 mg/L) and modified King’s A agar (Peptone 20 g/L, dipotassium hydrogen phosphate 1.5 g/L, magnesium chloride 1.5 g/L, agar 15 g/L, and 5-Chloro-2-(2,4-dichlorophenoxy) phenol 25 mg/L). Plates were incubated at 28°C for 48 h. Subsequently, the plates were evaluated under UV light in order to detect fluorescent pigment production. Single colonies representative of phenotypical variety were streaked onto petri dishes containing King’s B agar; subsequently they were characterized physiologically and biochemically by the API 20 NE test (bioMérieux^®^), growth at 42°C and growth on 6%, 7%, and 8% NaCl. For molecular bacterial characterization, genomic DNA was extracted using the Wizard^®^ Genomic DNA purification kit (Promega). PCR-mediated 16S rDNA amplification products was performed using universal primers PA and PC5B (Table 1). The products were sequenced using the 3730xl DNA sequencer (Applied Biosystems). The nucleotide sequences were compared to the 16S rDNA sequence from the GenBank database using a nucleotide BLAST search. For those bacterial isolates whose 16S rDNA similarity level was below 98.6%, accurate discrimination in the identity of bacteria isolated was obtained using a second 16S rDNA amplification with internal primers (Table 1).

**TABLE 1:** Primers used in this study.

Primer	Primer sequence (5′- 3′)	References
*fD1*e	AGAGTTTGATCCTGGCTCAG	Weisburg et al., 1991
*rD1*	AAGGAGGTGATCCAGCC	Weisburg et al., 1991
*362f	CTCCTACGGGAGGCAGCAGT	Mena et al., 2006
*786f	AAGCGTGGGGAGCAAACAGG	Mena et al., 2006
*1203f	AGGTGGGGATGACGTCA	Mena et al., 2006

*Internal primers.

### *Ex-planta* Antibacterial Ability Against *Xcc* Assay

*Ex-planta* antibacterial assays were performed by the disk diffusion technique. Briefly, three strains of *Xcc* 306: (i) two strains isolated from citrus leaves with citrus canker symptoms at the Paraná state and (ii) our *Xcc* 306 culture collection strain were grown in nutrient broth (NB) up log phase (OD600 0.5–0.7). Then, these strains at a final concentration 10^8^ CFU mL^–1^ adjusted in 10 mM MgCl_2_ were streaked with a sterile swab over the entire surface (pad fashion) of plates of nutrient agar (NA). Subsequently, a sterile filter paper disk was soaked with a suspension of 5 μL at final concentration at 10^8^ CFU mL^–1^ of each isolated bacterium from soil samples. This concentration was adjusted from each isolated bacterial culture grown at OD600 = 1.5–1.8. Subsequently, this disk was placed on the surface of NA agar plates previously seeded in pad fashion with each strain of *Xcc* 306 (three replicates for every strain) and incubated at 29°C. Afterward, the plates were evaluated to measure the growth inhibition halos of *Xcc* at 24 h and 48 h. The assay was then repeated, using a crude cell-free extract instead of bacterial cells isolated from soil samples. The extracts were dissolved in dimethyl sulfoxide (DMSO) at final concentrations of 100 μg, 50 μg, and 25 μg. DMSO was used as negative control. The strain *Bacillus amyloliquefaciens* LE109 a recognized agent of biological control was used as positive control at same concentration of isolated bacteria. Plates were incubated at 29°C. After 24 and 48 h three replicates plates were evaluated for growth inhibition halos. All experiments were repeated at same conditions three times.

### *In planta* Antagonism Assays

*Citrus* × *limonia* was chosen as the susceptible host, because it exhibits higher sensitivity to *Xcc* 306 (Graham, 2001). The assays were performed under controlled growth conditions at the Plant Laboratory, Technology Department FCAV/Universidade Estadual Paulista, SP, Brazil. Two methods of Infection were performed on attached leaves: leaf infiltration with needleless syringes and spray (Viloria et al., 2004; Rybak et al., 2009). All plants were grown in a growth chamber maintained at 28°C and with a photoperiod of 16 h. The antagonist bacteria tested were only those that showed a higher ability for *Xcc* 306 growth inhibition on *ex planta* assays. All plants were the same age at the time of inoculation; fully expanded immature citrus leaves of similar age were infected by infiltration pressure with needleless syringes containing both *Xcc* 306 and antagonist bacterial isolates. Each strain of *Xcc* 306 and the antagonist bacterial strains were mixed just prior to infection. The final concentration was adjusted to 10^8^ CFU mL^–1^ in 10 mM MgCl_2_ for each strain. MgCl_2_ was inoculated as a negative control and *B. amyloliquefaciens* LE109 from our lab culture collection was inoculated as positive control at same concentration of antagonist bacterial isolates. In order to assess the innocuity of antagonist bacteria in a susceptible citrus host, each antagonist bacteria were infiltrated in citrus leaves. Any antagonist bacteria that displayed minimal signs of pathogenic behavior (i.e., leaf spots, blights, wilts or scabs) against citrus host were rejected from study. The infiltrated leaves were photographed 21 DPI (days post-inoculation). Canker lesions from five infiltrated leaves for every treatment were quantified, and the infected areas were calculated using IMAGEJ v. 1.48 (Schneider et al., 2012).

In order to resemble the natural infection route to citrus host by *Xcc*, the spray infection method in attached leaves described by Li and Wang (2012) was performed with modifications. Briefly, unripe and fully expanded leaves of each plant were sprayed on the abaxial surfaces with a mix solution of each antagonist bacterium and *Xcc* 306. The final concentration of these solutions was 10^7^ CFU mL^–1^ in 10 mM MgCl_2_ for each strain. A mix solution of *Xcc* and *B. amyloliquefaciens* LE109 from our lab culture collection was sprayed as positive BCAs at same conditions. Next infection, the plants were enclosed with plastic bags for 24 h to provide a high relative humidity (>90%) and to favor the opening of stomata for symptom development. The sprayed leaves were photographed 21 DPI. Canker lesions from five infiltrated leaves and five sprayed leaves were quantified 21 DPI.

Additionally, endophytic bacteria were isolated from infected citrus leaves at 3 and 7 DPI by plating in king’s B medium, to recover antagonist *Pseudomonas* bacteria. The recovered strains were identified using the API 20 NE kit and 16S rDNA PCR amplification as mention above.

### MIC and MBC Determination

In order to determine the Minimum Inhibitory Concentration (MIC) at which *Xcc* 306 growth is inhibited, cell-free crude extracts of antagonist bacteria which displayed bactericidal activity against the different strains of *Xcc* 306 *ex planta* was assayed. Briefly, overnight King’s B grown cell cultures of each isolated antagonist bacteria was used to inoculate fresh King’s B broth at a volume of 1:100, it was then incubated at 30°C with shaking (200 rpm) until an OD600 ∼1.8. Subsequently, cells were removed by centrifugation (13000 *g*, 15 min, 4°C) and filtered through a 0.22-μm-pore-size membrane to remove residual bacterial cells. The supernatant was extracted three times with 0.5 vol. ethyl acetate, this fraction was evaporated to dryness in rotary evaporator and the material was weighed and dissolved in DMSO. MICs of extracts were determined by the macro dilution broth method following standard CLSI (Clinical and Laboratory Standard Institute) Protocols. 10 μL at final concentration 5 × 10^5^ UFC/mL of each strain of *Xcc* 306 suspension were used to inoculate 1 mL of NB. Concentrations of 100, 50, and 25 μg/mL of each extract were added to tubes containing each strain *Xcc* 306 and then incubated at 30°C with shaking (200 rpm) for 48 h. Bacterial optical density of was measured 24 and 36 and 48 h and compared to the controls (1 mL of NB inoculated simply with 5 × 10^5^ UFC/mL of each strain of *Xcc* 306) under the same incubation conditions. Each experiment was performed in triplicate and repeated fivefold. Minimal Bactericidal Concentration (MBC) for each treatment was determined by subculturing each NA agar plates.

### Extraction, Purification and Characterization of Putative Bioactive Compounds

To analyze the bioactive compounds, present in the cell-free crude extracts from the antagonist bacterial strains infiltrated in citrus host, supernatant from overnight cell cultures (OD ≥ 1.8) was extracted three times with 0.5 vol. ethyl acetate. This fraction was evaporated to dryness in a rotary evaporator and the material was weighed and dissolved in DMSO. To purify these DMSO fractions LC-DAD-IT and LC-DAD- TOF analyses were performed using an UFLC instrument (Shimadzu, Japan). LC-DAD-MS/MS was acquired using an UFLC apparatus coupled with an Ion Trap Mass Spectrometer (amaZon SL, Billerica, MA, United States), while LC-DAD-TOF was conducted by UFLC equipped with an UltrOTOF (Bruker Daltonics, Billerica, MA, United States) mass spectrometer. Samples used in LC-DAD-IT and LC-DAD-TOF were submitted to high-resolution mass spectrometry analysis. Samples were introduced into the ESI source by syringe pump and analyzed by an ultrOTOFQ (Bruker Daltonics, Billerica, MA, United States) mass spectrometer. The putative identification of bioactive compounds was assisted by molecular networking, i.e., clustering of MS/MS spectra by cosine similarity (Watrous et al., 2012; Wang et al., 2016). The data were subjected to Spectral Networks, which includes MS-Cluster, followed by visualization in Cytoscape 2.8.3. Putative structures of some molecular species were proposed by MS/MS-based fragmentation patterns.

### Data Analysis

All assays were performed in triplicate and repeated in independent trials at least three times. All data are presented as mean values ± standard error of the mean. Statistical analysis of data was performed using one-way ANOVA with *post hoc* testing (Bonferroni) using SPSS STATISTICS DESKTOP, v. 22.0 software (IBM). A significance level of 0.05 was considered for all analyses.

## Results

### Metagenomic Sequencing and Functional Screening

Using Illumina’s HiscanSQ platform for sequencing, an average of 28.6 million raw sequence reads were obtained from each soil sample. After quality assessment and trimming were produced an average 26.1 millions of reads for sample. The study was composed by 6 metagenomes identified as follows: H1A, H2A, H3A, H1S, H2S, H3S. The functional screening on the MG-RAST metagenomics analysis server, shown the presence of genes encoding for metabolites with antibacterial activity specially phenazines, it which were related with genomes of Pseudomonas bacteria evidenced in a previous study. The metagenomic data can be found at: https://www.mg-rast.org/linkin.cgi?project=mgp15294.

### Isolation and Characterization of *Pseudomonas*

A total of 37 isolates with distinct colony phenotypes (including fluorescent pigment production under UV light) were recovered in King’s A and King’s B media culture from the 12 soil samples. All isolates were screened for their ability to inhibit the growth of *Xcc* 306. Bacteria that displayed any antibacterial activity against *Xcc* 306 were identified as *Pseudomonas* species by molecular, biochemical and physiological methods (Table 2). It should be noted that based in 16S rDNA gene sequences, *Pseudomonas sp. JS2* identified as *P. entomophila* is closely related to *Pseudomonas monteilii* and *Pseudomonas mosselii* species i.e., 99.7 and 99.8% similarity (Mulet et al., 2012), for this reason, additional physiological and biochemical characteristics were tested by API 20NE, fluorescent pigment production and 42°C, 6%NaCl, 7% NaCl, 8%NaCl growth. Different test such as urease, gelatinase activity and others mention before aimed to evaluated phenotypic traits, which become in valuable tools that complement genotypic characterization, in order to get more discriminated classification at specie level (Table 3).

**TABLE 2:** Antagonist *Pseudomonas* bacteria isolated from soil samples.

Isolate	Closest species type strain	GenBank accession no	16S rDNA similarity (%)	Antagonist ability assay^a^
*Pseudomonas* sp. JS1	*Pseudomonas donghuensis* strain HYS	NR136501.1	99	+++
*Pseudomonas* sp. JS2	*Pseudomonas entomophila* strain L48	NR102854.1	99	+++
*Pseudomonas* sp. JS3	*Pseudomonas monteilii* strain CIP 104883	NR024910.1	99	+
*Pseudomonas* sp. JS4	*Pseudomonas oryzihabitans* strain L-1	NR025881.1	99	++
*Pseudomonas* sp. JS5	*Pseudomonas taiwanensis* strain BCRC 17751	NR116172.1	99	+++
*Pseudomonas* sp. JS6	*Pseudomonas nitroreducens* strain IAM 1439	NR042435.1	98	++
*Pseudomonas* sp. JS7	*Pseudomonas mosselii* strain CFML 90-83	NR024924.1	99	++
*Pseudomonas* sp. JS8	*Pseudomonas putida* strain NBRC 14164	NR11365.1	98	+

*^*a*^Semiquantitative measurements.*

**TABLE 3:** Differential phenotypic traits of isolated *Pseudomonas* strains.

Characteristics	JS1	JS2	JS3	JS4	JS5	JS6	JS7	JS8
**Production fluorescent pigments**								
Agar King’B	+	+	−	−	+	−	+	+
Agar King’A	−	−	−	−	−	−	−	−
**Growth**								
42°C	−	+	−	−	+	−	−	−
6% NaCl	−	+	+	+	+	+	+	−
7% NaCl	−	−	−	−	−	−	−	−
8% NaCl	−	−	−	−	−	−	−	−
**API 20 NE**								
Reduction of nitrate	+	−	−	−	−	+	−	−
Presence of arginine dihydrolase	−	+	+	−	+	−	+	+
Urease	−	−	−	+	−	+	+	−
Hydrolysis of gelatin	+	+	−	−	−	−	+	−
L-Arabinose 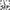	+	−	+	+	+	−	−	−
D-Mannose 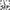	+	+	−	+	−	−	+	−
D-Manitol	+	+	−	+	−	−	+	−
N-acetyl-glucosamine	+	+	−	−	−	+	+	−
Phenylacetic acid	+	+	+	−	+	−	+	+

### *Ex-planta* Assays: MIC and MBC for *Xanthomonas citri* subsp. *citri* 306 Strains

**FIGURE 1:**
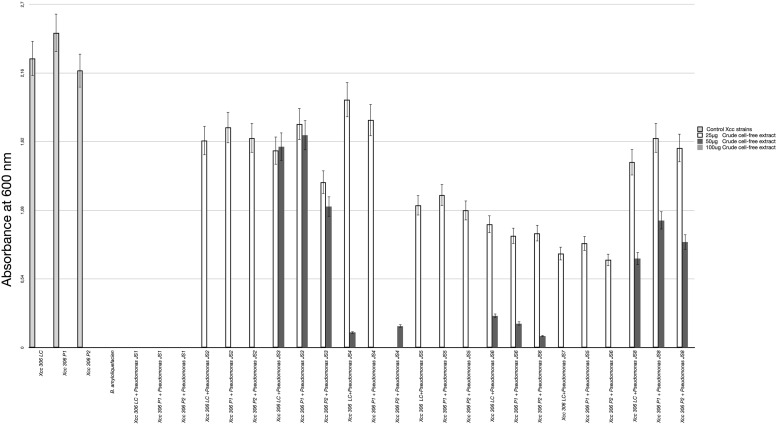
*Xcc* 306 strains growth at 36 h on incubation with different concentrations of crude cell-free extract from each isolated Pseudomonas strains. Values given are the means and error bars represent standard deviation of measurements. The assays were repeated three times in five independent experiments for each antagonistic strain, yielding similar results. Only one representative result is presented in the figure. *Xcc* 306 LC is laboratory culture collection strain. *Xcc* 306 P1 is the strain isolated from citrus cultivar 1 with citrus canker symptoms at Paraná state. Brazil and *Xcc* 306 P2 is the strain isolated from citrus cultivar 2 with citrus canker symptoms at Paraná state, Brazil.

All 37 bacterial isolates from soil samples were screened by the agar diffusion technique in their ability to inhibit the growth of *Xcc* 306 strains. Only 8 displayed any inhibition effect. These were characterized as Pseudomonads and classified according to the *Xcc* 306 growth inhibition halo as: high, medium, and low antagonists (Table 2). The antagonism assay by Pseudomonas isolated showed a size inhibition zone from 8 to 14 mm. The inhibition halo produce by *B. amyloliquefaciens* LE109 was 16 mm. This behavior was similar in the three *Xcc* 306 strains assessed. Size inhibition zones were also measured using crude bioactive cell-free extracts and were 0.4–1.5 fold higher (Supplementary Figure S1). The MIC was defined as the lowest crude extract concentration at which every strain of *Xcc* 306 shows no growth after 36 h of incubation. *Pseudomonas* sp. JS1 JS2, JS5, JS7 JS3, JS4, and JS8 all displayed antibacterial activity against *Xcc* 306 ranging from 25 to 100 μg of crude extract. The MIC and MBCs for one of the isolates, *Pseudomonas* sp. JS1 identified as *Pseudomonas donghuensis* was 25 μg. This was the strongest *ex-planta* antibacterial effect. MBCs for isolates JS2, JS5, and JS7 were 50 μg, while for JS3, JS4, and JS8 they were 100 μg. The MIC and MBC for *B. amyloliquefaciens* LE109 were 25 μg (Figure 1).

### *In planta* Antibacterial Activity Against *Xcc* 306

**FIGURE 2:**
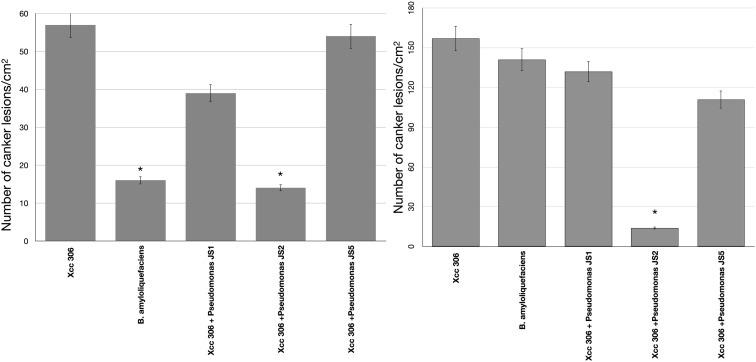
*Pseudomonas* antibacterial activity against *Xcc* 306 *in planta* assays. Quantification of canker lesions in *Citrus × limonia* leaves at 21 days post-inoculation. **(A)** Leaves inoculated by pressure infiltration with a mixture of antagonist *Pseudomonas* bacteria and *Xcc* 306 strains (10^8^ CFU mL^–1^ for each strain). **(B)** Leaves inoculated by spraying a mixture of antagonist *Pseudomonas* bacteria and *Xcc* 306 (10^7^ CFU mL^–1^ for each strain). Values given are the means and error bars represent standard deviation of measurements. Values marked with an asterisk are significantly different from *Xcc* 306 alone at *P* < 0.05 using one-way ANOVA with *post hoc* test (Bonferroni) with SPSS STATISTICS DESKTOP software, v. 22.0. The assays were repeated three times in five independent experiments for each antagonistic strain, yielding similar results. Only one representative result is presented in the figure.

**FIGURE 3:**
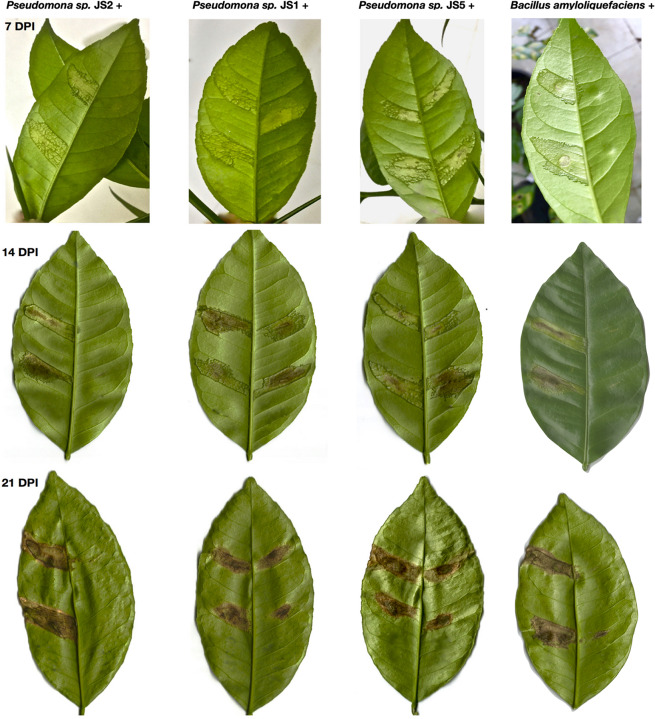
Reduction in the severity of citrus canker disease by the action of antagonist *Pseudomonas* bacteria isolated from soil samples. *Citrus × limonia* leaves infected by infiltration pressure at 7, 14, and 21 DPI. Right side of leaf: antagonist *Pseudomonas* isolated plus *Xcc* 306. Left side of leaf: *Xcc* 306 (10^8^ CFU mL^–1^). Both bacteria were co-infiltrated at equal concentration (10^8^ CFU mL^–1^). Bacterial strains were mixed just prior to infection. The assays were repeated three times with three plants each time, yielding similar results. Only one representative result is presented in the figure.

**FIGURE 4:**
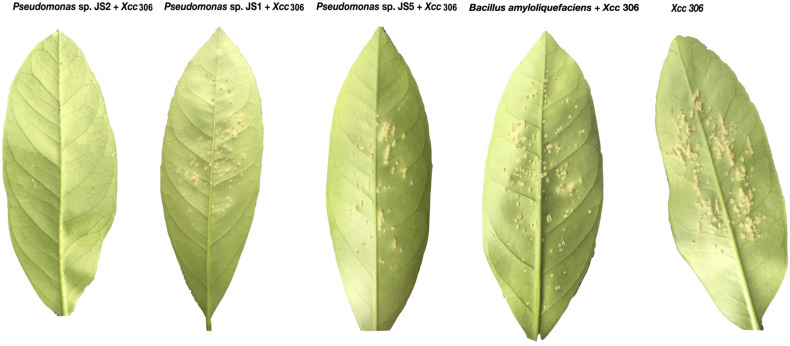
Reduction in the severity of citrus canker disease by the action of antagonist *Pseudomonas* bacteria isolated from soil samples. Spray inoculation on the abaxial side of *Citrus × limonia* leaves. both bacteria were co- inoculated at a concentration of 10^7^ CFU mL^–1^ Inoculated leaves were photographed at 21 days post-inoculation.

To verify the antibacterial effect of isolated *Pseudomonas* against the *Xcc* 306 strains infecting a highly susceptible host, as well to measure its effect on citrus canker severity, *Citrus x limonia* leaves were infected with a mixture of the antagonist Pseudomonads and *Xcc* 306 at equal population density by the infiltration pressure and spray methods. *Citrus × limonia* plants were grown under controlled conditions and citrus canker lesions were quantified at 21 DPI. None of antagonist bacteria exhibited virulence against citrus host (data not shown). Our findings show that only the *Pseudomonas* sp. JS2 displayed a clear citrus canker reducing effect (Figures 2–4). B. *amyloliquefaciens* don’t displayed any effect in reducing citrus canker symptoms in the leave infected by spray methods. Unlike, in leaves infected by pression infiltration method in which a reduction on canker was evidenced. Results of bacterial recovery assays from infected plants showed that only *Pseudomonas* sp. JS2 grew in king’s B medium at 3 and 7 DPI. Other Pseudomonad isolates, in particular *Pseudomonas* sp. JS1 which displayed a higher *ex planta* antibacterial activity against *Xcc* 306 was not recovered from infected leaves at 3 and 7 DPI. *B. amyloliquefaciens* was only recovered from leaves infected by infiltration method not from leave infected by spray method.

### Identification of Putative Bioactive Compound

Identification of putative bioactive compound using the molecular networking for 3 analyzed bacterial strains (including those that did not shown a significant effect in citrus canker severity reduction) has revealed a clearly defined cluster of small cyclic peptides that could be partially responsible for the antibacterial activity. These were identified as members of diketopiperazines (DKPs) molecule family.

## Discussion

In nature most microbes face continuous competition for resources. Potential successful bacterial competitors might therefore be expected to encode an array of mechanisms allowing them to emerge and dominate particular bacterial populations. Bacteria belonging to the genus *Pseudomonas* have a highly variable genome size from 3.7 to 7.1 Mb, containing more than 6390 predicted genes (Singh et al., 2016). This is consistent with their capacity to produce an extensive repertoire of organic compounds including simple carbohydrates as well as more complex biomolecules such as biosurfactants, insecticides, phytotoxic compounds, antibiotics and siderophores, which enable them to compete in highly diverse ecological niches. Understanding this sophisticated metabolic repertoire and its roles in the diverse environmental niches, will provide invaluable information which could be used to improve growth promoting activities and biological control of phytopathogens in commercial crops. Biological control in citrus canker disease has been addressed by different approaches. Several studies have been show that bacterial species from genera *Bacillus* (*Bacillus subtilis* and *Bacillus polymyxa)* and *Pseudomonas* (*P. fluorescens* and *Pseudomonas oryzihabitans*) isolated from citrus phylloplane display a great *ex planta* antibacterial activity against *Xcc*. However, when this bacterial antagonist were inoculated by spray method in citrus host, the reduction of symptoms was lesser than expected (Kalita et al., 1996; Gade and Lad, 2018). Endophytic bacteria *Bacillus thuringiensis* TbL-22 and TbL- 26 isolated from gymnospermic and angiospermic plants were inoculated by pression infiltration methods in orange leaves, this bacteria reduce canker symptoms (Islam et al., 2019). Perhaps, the greatest drawback in the biological control of citrus canker is the successful fitness to phyllosphere niche by BCAs. In this study by metagenomic and culture dependent approaches we isolated 8 bacterial strains from soil samples of São Paulo Zoo Park farm which belong to the *Pseudomonas* genus and are able to inhibit *ex planta Xcc* 306 growth. Since, the totality of *Pseudomonas* species screened for their ability to inhibit *ex planta* the *Xcc* 306 growth were isolated from soil samples together with the hallmark metabolic plasticity of *Pseudomonas* genus, it is reasonable to think that successful fitness to a very different ecological niche i.e., mesophyll tissue in plant leaves, must not represent an enormous challenge for these bacteria. Remarkably, only the strain *Pseudomonas* sp. JS2 identified as putative *P. entomophila* has been recovered from infected plants, moreover, *Pseudomonas* sp. JS2 was the most successful treatment reducing almost completely citrus canker symptoms in highest susceptible citrus hosts. Since their initial isolation, much of the research developed on *P. entomophila* has focused on the study of interaction with the insect host to understand its entomopathogenicity (Vodovar et al., 2005; Chakrabarti et al., 2012). There are few studies which have focused on its other properties, examples include a *P. entomophila* strain isolated from the rhizosphere of red pepper in South Korea displays a capacity to act as a plant growth promoter by providing resistance to several stress factors (Kamala-Kannan et al., 2010) and the results of a metagenomic study of Artic soil samples highly contaminated with diesel fuel, where *P. entomophila* was repeatedly identified as member of local microbiota, raising the possibility of a potential application in bioremediation processes (Yergeau et al., 2012). Dissimilar behavior was shown by *Pseudomonas* sp. JS1 identified as *P. donghuensis* and *Pseudomonas* sp. JS5 identified as *Pseudomonas taiwanensis*, which despite displaying a high inhibitory effect over *Xcc* 306 growth in the *ex planta assay* (Figure 1), do not show significant reduction of citrus canker symptoms in susceptible host (*in planta*). Indeed, *Pseudomonas* sp. JS1 and *Pseudomonas* sp. JS5 could not be recovered from *Xcc* 306 coinfected tissues. These facts strongly suggest an incompatibility relationship between *Pseudomonas* sp. JS1, *Pseudomonas* sp. JS5 and *Citrus × limonia* plants, it which evidencing the difficulty of these bacteria to fitness as successful endophytes in citrus leaves. One possible explanation could be that, in contrast to *Pseudomonas* sp. JS2, *Pseudomonas* sp. JS1, and *Pseudomonas* sp. JS5 are devoid of T6SS (Type VI Secretion System) (Sarris and Scoulica, 2011). Although T6SS was initially identified in the pathogenic bacteria *Vibrio cholerae* and *Pseudomonas aeruginosa* (Mougous et al., 2006), it later was identified in non-pathogenic bacteria (Marchi et al., 2013). Analysis of T6SS in plant-microbe interactions established a role of this secretion system in an endophytic lifestyle (Reinhold-Hurek and Hurek, 2011). Possible additional explanations for the refractory colonization of mesophyll tissue in citrus leaves by *Pseudomonas* sp. JS1 and *Pseudomonas* sp. JS5, could be: (i) some of their structural components can act as Pathogen Associated Molecular Patterns (PAMPs) triggering an immune response in the citrus host, causing a failure in bacterial establishment and multiplication within the host, however, this remains speculative, (ii) The apoplast low pH could efficiently restricts bacterial multiplication. *P. entomophila* can grow in hostile environments as insect midgut where the pH is lower than 3.0, which providing to *P. entomophila* with an additional advantage to successfully colonize the mesophyll tissue.

HPLC-Ion Trap-MS assays assisted by TOF-MS, MS/MS-based fragmentation and molecular networking unveiled the chemical nature of putative compound with antibacterial activity as a cyclodipeptide known as diketopiperazines (DKPs). DKPs are produced by a large range of gram negative bacteria. Important biological activities attributed to DKPs comprising: antiviral (Sinha et al., 2004) antifungal, antibacterial (Lee et al., 2010), bacterial quorum quenchers and antiprion (Bolognesi et al., 2010). DKPs are biosynthesized by dedicated non-ribosomal peptide synthetases (NRPSs). However, an alternative route that also employs NRPSs, synthetizes them as truncated side products during the synthesis of longer peptides, as has been documented in *E. coli* (Gruenewald et al., 2004). DKPs produced by Pseudomonads are cyclo(L-Leu-L-Pro), cyclo(L-Phe-L-Pro), cyclo(L-Pro-L-Tyr), and cyclo(L-Leu-L-VaL) (Holden et al., 1999). DKPs are recognized antagonists of N-AHL-dependent quorum sensing. Since quorum sensing in *Xcc* 306 is mediated by molecule *cis*-11-methyl-2- dodecenoic acid belonging to DSF autoinducer family. We discard that reduction of citrus canker severity was due to perturbation of cell-cell communication system by DKPs. It was recently documented that disruption of DSF quorum sensing in *Xcc* 306 by Pseudomonads employ a quorum quencher mechanism different than DKPs, which was based on modification of DSF molecule via addition of sugar moiety by UDP-sugar transferase enzymes (Caicedo et al., 2016). Additionally, *P. entomophila* harbors five NRPS gene clusters coding for NRPSs, which are responsible for synthesizing at least three different lipopeptides and a polyketide of unknown function (Vodovar et al., 2006). Because the DKPs production has been identified in the concentrated crude cell-free extracts from all three bacterial isolated infiltrated in the citrus host, even in those strains that don’t reduce citrus canker lesions, we suggest that significantly reduction of canker disease severity by *Pseudomonas* sp. JS2 *in planta* is not exclusively by DKP action, instead a collective action of T6SS, DKPs, and NPRSs product, could be necessaries for the successful biological control of citrus canker disease. Finally, since their initial isolation, much of the research developed on *P. entomophila* has focused on the study of interaction with the insect host to understand its entomopathogenicity. There are few studies which have focused on its other properties, examples include a *P. entomophila* strain isolated from the rhizosphere of red pepper in South Korea displays a capacity to act as a plant growth promoter by providing resistance to several stress factors (Kamala-Kannan et al., 2010) and the results of a metagenomic study of Artic soil samples highly contaminated with diesel fuel, where *P. entomophila* was repeatedly identified as member of local microbiota, raising the possibility of a potential application in bioremediation processes (Yergeau et al., 2012).

The entomopathogenic ability of *P. entomophila* against insects of hemiptera order has not yet been confirmed, however, due to its strong entomopathogenicity this bacterium could be a promissory biocontrol agent in vector borne diseases as variegated chlorosis and citrus greening, which are serious threat to citrus crops.

## Data Availability Statement

The datasets generated for this study can be found in the https://www.mg-rast.org/linkin.cgi?project=mgp15294.

## Author Contributions

SV, JF, JC, and LA contributed to the concept and design of this research. SV conducted the bioinformatic analyses. SV and JC isolated and characterized the *Pseudomonas* bacteria from soil samples. SV and JC contributed to the antagonist assays *in* and *ex planta* and drafted the manuscript. All authors critically revised it and agreed to all aspects of the work presented.

## Conflict of Interest

The authors declare that the research was conducted in the absence of any commercial or financial relationships that could be construed as a potential conflict of interest.

## Supplementary Material

FIGURE S1 Ex-planta antibacterial activity assays. **(A–C)** Show agar diffusion technique antagonist assays between cell free crude extract of *Pseudomonas* bacteria (25 μg, 50 μg and 100 μg concentrations) and *Xcc*. **(D)** Show MIC determination between one *Pseudomonas* strain (25 μg, 50 μg and 100 μg concentrations) that displayed a moderate antagonist activity against *Xcc*.
