# Supplementary figures and images for: Bactericidal Effect of Entomopathogenic Bacterium Pseudomonas entomophila Against Xanthomonas citri Reduces Citrus Canker Disease Severity
Source: Front Microbiol. 2020 Jun 24;11:1431. doi: 10.3389/fmicb.2020.01431 (PMC7327231; doi:10.3389/fmicb.2020.01431)

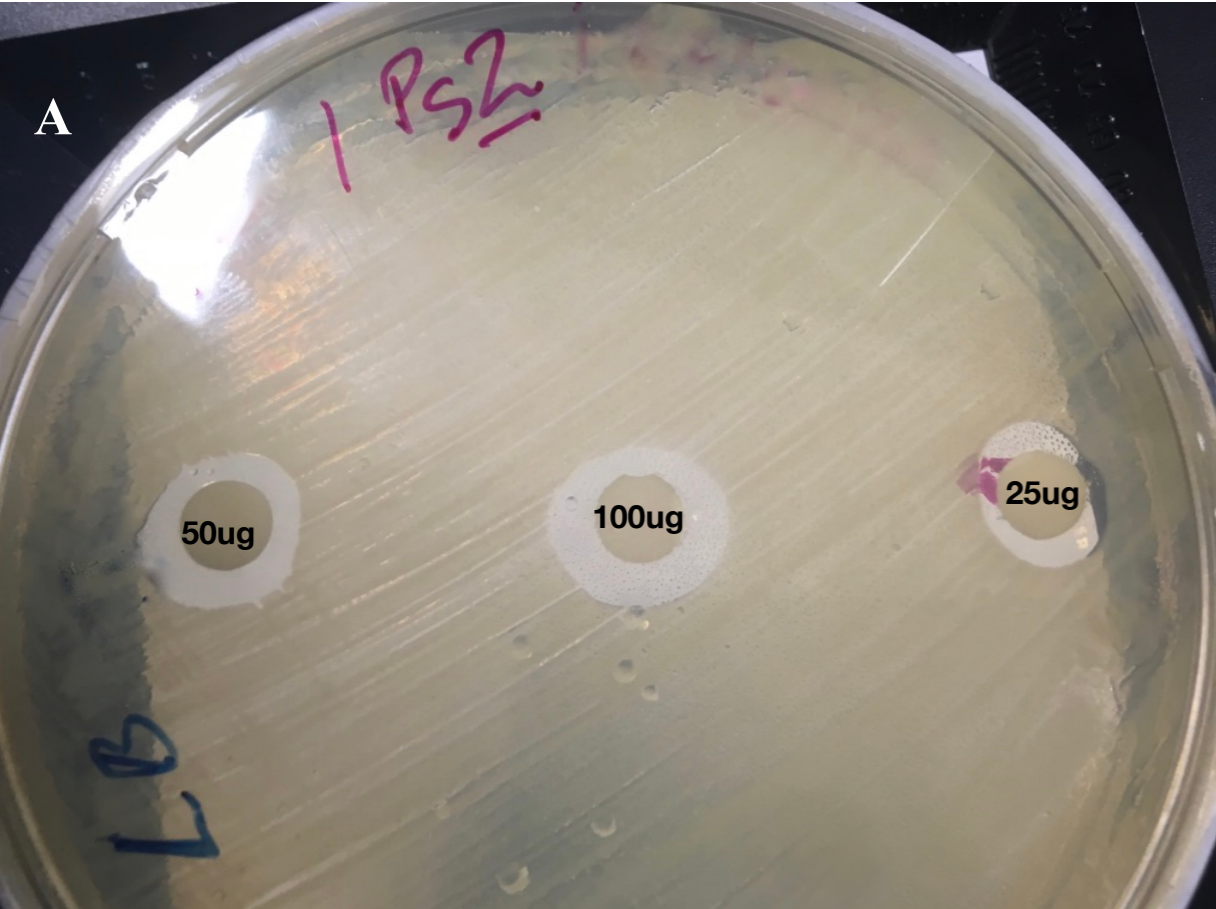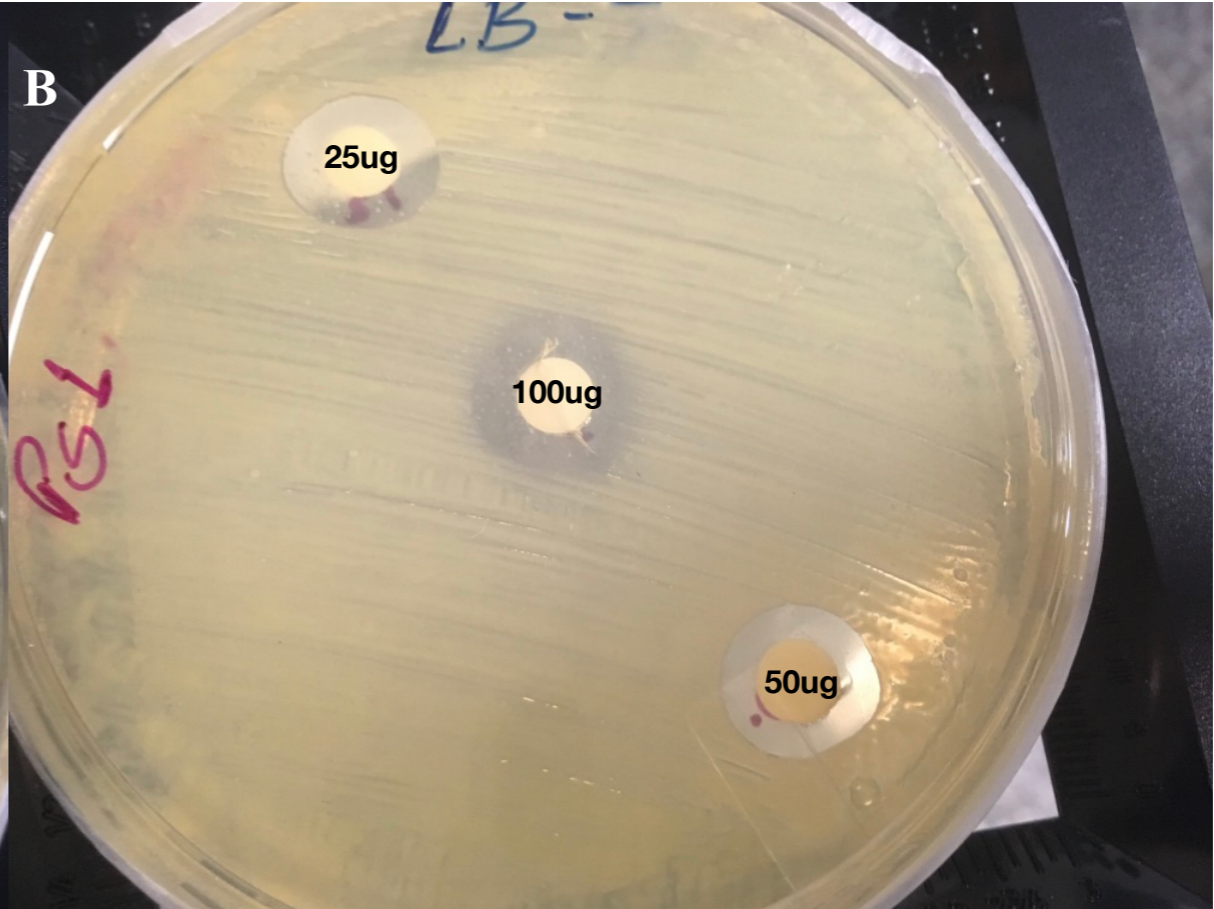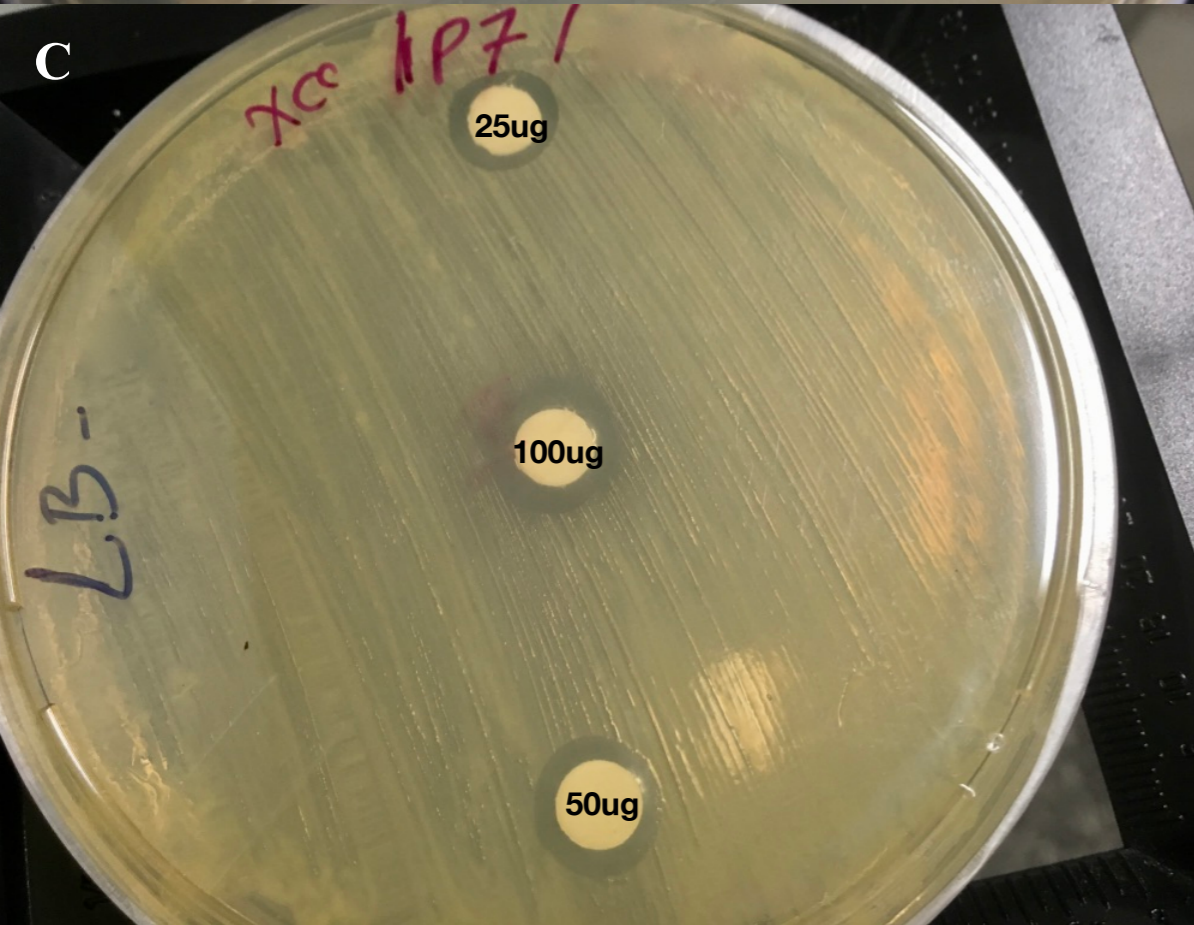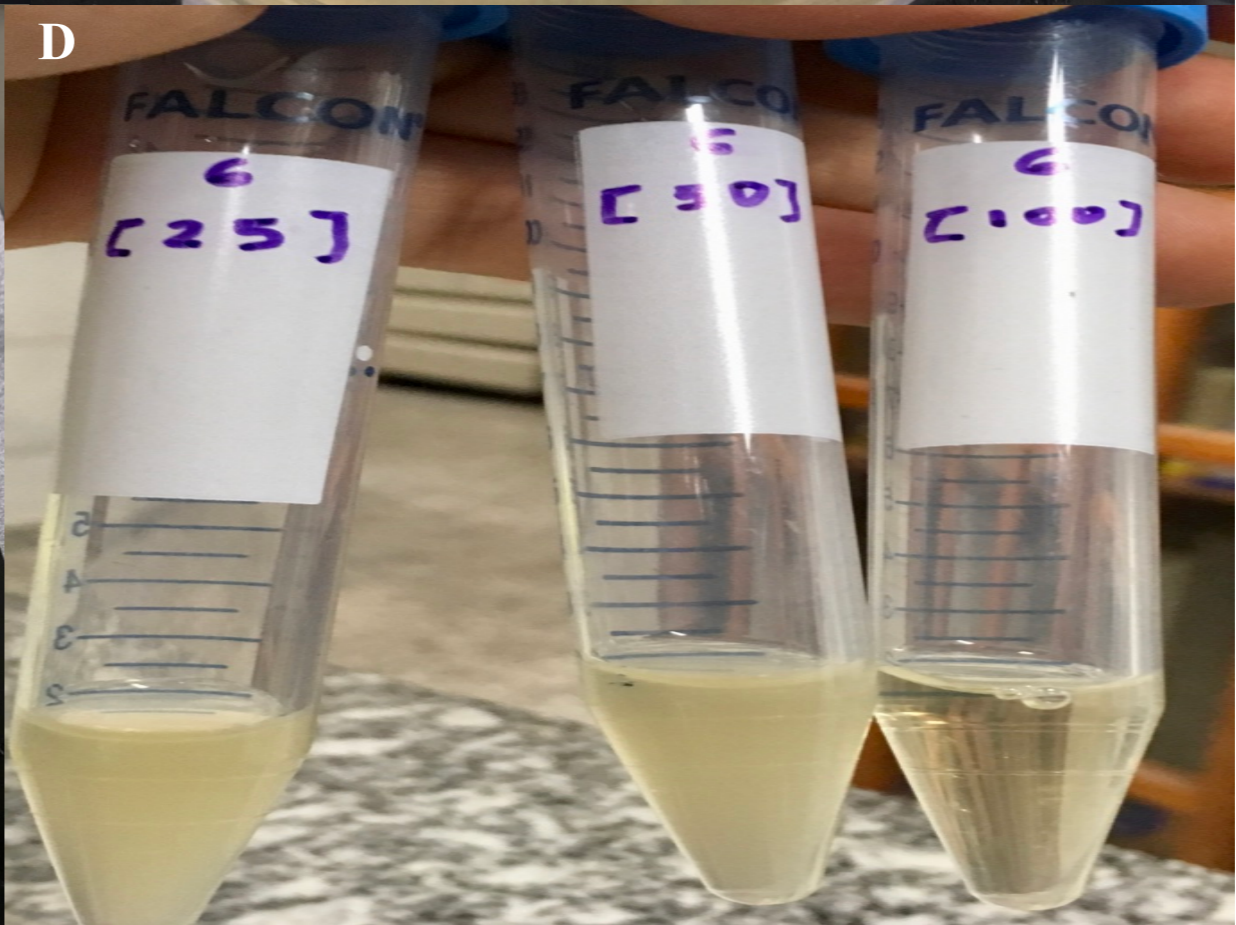

Supplement: FIGURE S1 — Ex-planta antibacterial activity assays. (A–C) Show agar diffusion technique antagonist assays between cell free crude extract of Pseudomonas bacteria (25 μg, 50 μg and 100 μg concentrations) and Xcc. (D) Show MIC determination between one Pseudomonas strain (25 μg, 50 μg and 100 μg concentrations) that displayed a moderate antagonist activity against Xcc. [file Data_Sheet_1.PDF]
